# The neck–shaft angle: an update on reference values and associated factors

**DOI:** 10.1080/17453674.2019.1690873

**Published:** 2019-11-18

**Authors:** Cornelius S Fischer, Jens-Peter Kühn, Henry Völzke, Till Ittermann, Denis Gümbel, Richard Kasch, Lyubomir Haralambiev, René Laqua, Peter Hinz, Jörn Lange

**Affiliations:** a Clinic of Trauma, Reconstructive Surgery and Rehabilitation Medicine, University Medicine Greifswald, Greifswald;; b Institute of Diagnostic Radiology and Neuroradiology, University Medicine Greifswald, Greifswald;; c Institute for Community Medicine, Ernst-Moritz-Arndt University of Greifswald, Greifswald;; d Center for Orthopedics, Trauma Surgery and Rehabilitation Medicine; Clinic and Outpatient Clinic for Orthopedics and Orthopedic Surgery, University Medicine Greifswald, Greifswald;; e Department of Radiology, Städtisches Krankenhaus Kiel, Kiel;; f Clinic and Policlinic of Radiology, University Hospital Dresden, Carl-Gustav-Carus University, Dresden, Germany

## Abstract

Background and purpose — The neck–shaft angle (NSA) is valuable for diagnostics and therapy of the hip, but current reference values derive mostly from studies on anatomic specimens, small cohorts, or are hospital-based. Moreover, associated factors such as age, sex, or anthropometric data have rarely been considered. Therefore, we determined associated factors for NSA and reassessed the historical reference values in a general adult population.

Methods — NSAs on both sides of 3,226 volunteers from the population-based Study of Health in Pomerania (SHIP) were measured with MRI. SHIP drew a representative sample of the population of Pomerania (northeastern Germany). NSAs were compared with sex, age, and anthropometric data by bivariable linear regression models. Reference values were assessed by quantile regressions for 2.5th and 97.5th percentiles.

Results — The mean NSA was 127° (SD 7), while men had a lower NSA than women (95% confidence interval [CI] 0.4°–1.4°). The reference range was 114°–140°. Age was inversely associated with NSA (CI –0.2 to –0.1). Body height was positively associated with the NSA, while BMI and waist circumference showed a negative association. There was no association between body weight and NSA.

Interpretation — The historical lower limit of 120° might be too high, so the radiological prevalence of hip pathology might have been overestimated. The previously reported influence of age, sex, and body height on the NSA has been confirmed.

The femoral neck–shaft angle (NSA) or centrum-collum-diaphyseal angle has long served as an important measure to quantify the inclination between the femoral neck and the femoral shaft. Due to its importance in diagnostics for many pathologies of the proximal femur and resultant therapy, e.g., corrective osteotomy (Srisaarn et al. 2019), the NSA is still frequently used by surgeons. However, the currently published reference ranges were mostly based on anatomic specimens, small cohorts (Boese et al. 2016), or originated from hospital-based studies.

Varying ranges have been described as reference ranges, between 120° and 140° in CT images and radiographs. Thresholds for Coxa vara range between < 120° (Beall et al. 2008, Dolan et al. 2011, Boese et al. 2015) and < 130° (Morvan et al. 2013), and for Coxa valga from ≥ 135° (Beall et al. 2008, Coskun Benlidayi et al. 2015) to > 140° (Dolan et al. 2011, Morvan et al. 2013). Those thresholds were mostly used in older studies on hip pathology (Tönnis and Heinecke 1999, Lequesne et al. 1964). Because of changing lifestyles, people are older, heavier, and taller now than they were several decades ago. Therefore, a reevaluation of the above-mentioned thresholds is motivated.

Possible sex-based differences in the NSA have been investigated, with divergent results. Some documented higher NSA for men (Elbuken et al. 2012, Nissen et al. 2005, Mitra et al. 2014), others for women (Boissonneault et al. 2014), or no sex difference at all was found (Doherty et al. 2008, Gilligan et al. 2013). The decrease of NSA from childhood to adulthood is generally accepted (Anderson and Trinkaus 1998, Beall et al. 2008, Boese et al. 2016). Reduction in NSA with increasing age has been described (Beall et al. 2008, Boese et al. 2015), but the opposite results have also been published (Anderson and Trinkaus 1998, Nissen et al. 2005, Doherty et al. 2008, Elbuken et al. 2012, Gilligan et al. 2013). Given the variety of physiological factors influencing NSA, adjusted reference values might be advantageous for more precise interpretation.

Therefore, we determined up-to-date NSA reference ranges and assessed associations between the NSA, sex, age and anthropometric data (weight, height, BMI, and waist circumference). Moreover, reference values adjusted to the already described associations with sex and age were calculated. 

## Methods

### Design and sample

The Study of Health in Pomerania (SHIP) (Völzke et al. 2011) is an ongoing population-based project. It consists of 2 independent cohorts, SHIP and SHIP-Trend. To ensure a representative cohort, participants were recruited randomly from official resident registry office files as a sample of the population from a defined region in northeastern Germany (northern and eastern Pomerania) and stratified by sex, age, and city of residence.

In 1997, 6,265 eligible adults were chosen for the baseline assessment (SHIP-0) in which 4,308 (2,192 women) volunteers participated (response 68.8%). Between 1997 and 2001, the SHIP-0 examinations were performed. 2 follow-up examinations took place between 2002 and 2006 (SHIP-1; n = 3,300) and between 2008 and 2012 (SHIP-2; n = 2,333). For the second cohort (SHIP-Trend), a stratified sample (n = 8,016) was drawn in 2008 with the same stratification variables used. 4,420 (2,275 women) participants were then examined (response 50%) (Völzke et al. 2011). The invitation procedure consisted of 3 written invitations, phone calls, and 1 personal contact.

As an associated project of SHIP, all participants with a complete hip protocol were included in the MRI examination from SHIP-2 and SHIP-Trend. Overall 3,317 out of 6,753 volunteers (SHIP-2 and SHIP-Trend) participated in the MRI examination. Dropouts were for instance caused by claustrophobia, metal implants, or personal reasons. Of 3,317 potential participants, 34 interrupted their examination due to acute problems. Furthermore, 57 of 3,283 completed pelvic MRIs had to be excluded because of missing data (36), total hip arthroplasty (18), extreme deformity (2), or suboptimal quality (1) (Fischer et al. 2018). MRIs of 3,226 volunteers with a mean age of 53 years (SD 14) with no difference between sexes were adequate for the current study. Further information on recruitment and selection has been published previously (Völzke et al. 2011, Fischer et al. 2018).

### MRI protocol

As a part of the standardized whole-body MRI, pelvic MRI was performed in a 1.5-Tesla MR scanner (Magnetom Avanto; Siemens Medical Systems, Erlangen, Germany). All MRI examinations were performed in a supine position by 4 trained technicians in a standardized manner.

The NSA was assessed by using a coronal turbo inversion recovery magnitude sequence (repetition time 4,891 msec; echo time 67 msec; flip angle 180°; voxel size 2.1 Ч 1.6 Ч 5.0 mm; scan time 2:25 minutes) and an axial pelvic proton density-turbo spin echo-fat saturation sequence (repetition time 3,230 msec; echo time 34 msec; flip angle 180 degrees; voxel size 1.6 Ч 1.6 Ч 3.0 mm; scan time 2:43 minutes).

### Image analysis

**Figure 1.**
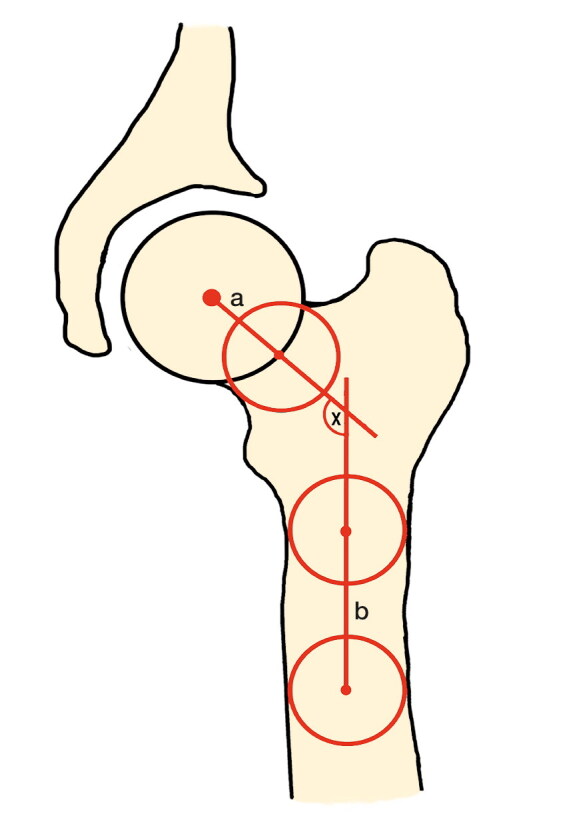
The neck–shaft angle (x) is composed of a and b; a = femoral neck axis, b = femoral shaft axis.

All measurements were performed by a trained observer, blinded to all information about the volunteers, using OsiriX version 5.8.5 (PIXMEO; Bernex, Switzerland). The NSA was measured on a coronal planar image through the center of the femoral head, which was identified by using axial slices simultaneously. It is defined by the femoral neck axis and the femoral shaft axis (Figure 1). The femoral neck axis was formed by connecting the center of the femoral head and the center of the femoral neck. Both center points were assessed through the center of a best-fitting circle. The femoral shaft axis was detected by two midpoints in the diaphysis of the femur. The midpoints were determined by the center of a best-fitting circle as well (Beall et al. 2008).  

### Statistics

For reliability assessment, 25 cases were measured twice by one examiner (CF). These cases were measured again by another examiner (JL). Intrareader and interreader variability (Bland and Altman plots) were between –0.31% (SD1.37) and 0.31% (SD 1.69) (mean difference), implying good reliability.

Descriptive statistics such as mean values, standard deviations (SD), ranges, and percentiles were used to describe the cohort. For numerical variables, categorical variables were tested with the chi-square test. All reported p-values were 2-tailed. P-values less than 0.05 were considered statistically significant. The correlation between sides (left and right) was calculated with Spearman’s correlation coefficient.

Associations with demographic and anamnestic data, including possible interactions between the parameters, were assessed by linear regression analysis. Fractional polynomials (FP) were tested for potential non-linear associations between age and NSA. Interactions between age and sex were tested, with p < 0.1 considered statistically significant. Stratified by sex, age-specific upper and lower reference limits were calculated by quantile regressions for the 2.5th and 97.5th percentile. Moreover, reference values were calculated by the 95% reference interval (mean ± 1.96 Ч SD). All statistical analyses were performed using Stata 14.1 (StataCorp, College Station, TX, USA).

### Ethics, funding, and potential conflicts of interests

Each participant gave written informed consent and the local ethics commission approved the SHIP study (BB 39/08, 19.06.2008). This study was performed as a SHIP-associated project (SHIP/2015/145/D) at University Medicine Greifswald, Germany. The SHIP study is part of the Community Medicine Research Net of the University of Greifswald, Germany, which is funded by the Federal Ministry of Education and Research (grant No. 03ZIK012), the Ministry of Cultural Affairs, as well as the Social Ministry of the Federal State of Mecklenburg-West Pomerania. MR imaging was supported by the Federal State of Mecklenburg-Vorpommern, the Federal Ministry of Education and Research, and a joint grant from Siemens Healthcare, Erlangen, Germany. Each author certifies that no competing interests exist. 

## Results

1,639 of the 3,226 participants were female. The mean age was 53 (SD 14) years and no differences in age were observed between the sexes (Table 1). The mean values of body height, body weight, BMI, and waist circumference were higher in men.

**Table 1.** Study cohort characteristics. Data are presented as mean (SD) [range]

Factor	Total (n = 3,226)	Men (n = 1,587)	Women (n = 1,639)
Age	53 (14) [21–90]	53 (14) [21–90]	52 (13) [21–88]
Weight, kg	80 (15) [44–143]	88 (13) [53–143]	73 (13) [44–126]
Height, cm	170 (9) [146–202]	177 (7) [156–202]	164 (6) [146–189]
BMI	28 (4) [17–48]	28 (4) [18–42]	27 (5) [17–48]
Waist, cm^a^	90 (13) [55–145]	96 (11) [66–145]	84 (12) [54–122]

a Waist circumference (2 women and 1 man missing).

The mean NSA for all 6,452 measured hips (left and right side) was 127° (SD 7, range 102°–151°). Right hip joints had a higher NSA than left hip joints. Additionally, women showed higher NSA than men on the left and the mean of both sides (Table 2).

**Table 2.** Descriptive results neck-shaft angle. Data are presented as mean (SD) [range]

Neck-shaft angle	Total^a^ (n = 6,452)	Men (n = 3,174)	Women (n = 3,278)
Right	127.7 (7.1) [96–153]	127.7 (7.1) [96–153]	127.9 (7.1) [99–152]
Left^b^	126.0 (7.4) [101–153]	125.3 (7.7) [101–151]	126.8 (7.1) [103–153]
Mean **^b,c^**	126.9 (6.7) [102–151]	126.5 (6.8) [102–151]	127.4 (6.6) [102–149]

a Paired t-test (total right vs. total left): p < 0.001.

b Sign test (men vs. women): p < 0.001.

c Mean of left and right side.

A correlation between left and right NSA values was documented (r = 0.7, p = 0.001). Additionally, a 0.9° (95% CI 0.4°–1.4°) higher NSA in women was detected. Furthermore, age was inversely associated with NSA (β –0.1; CI –0.2 to –0.1). The NSA decreases by 1.4° per decade. The reference range for the whole population was 114°–140°. Stratified by sex, the female reference range was 115°–140°, while the male range was 113°–140°. Age- and sex-adjusted reference values for women can be calculated by NSA = 125.78 – 0.195ЧЧЧage for the lower limit and NSA = 147.82 – 0.158ЧЧЧage for the upper limit. For men, the lower limit can be calculated by NSA = 120.18 – 0.128ЧЧЧage and the upper limit by NSA = 145.34 – 0.124ЧЧЧage.

**Figure 2.**
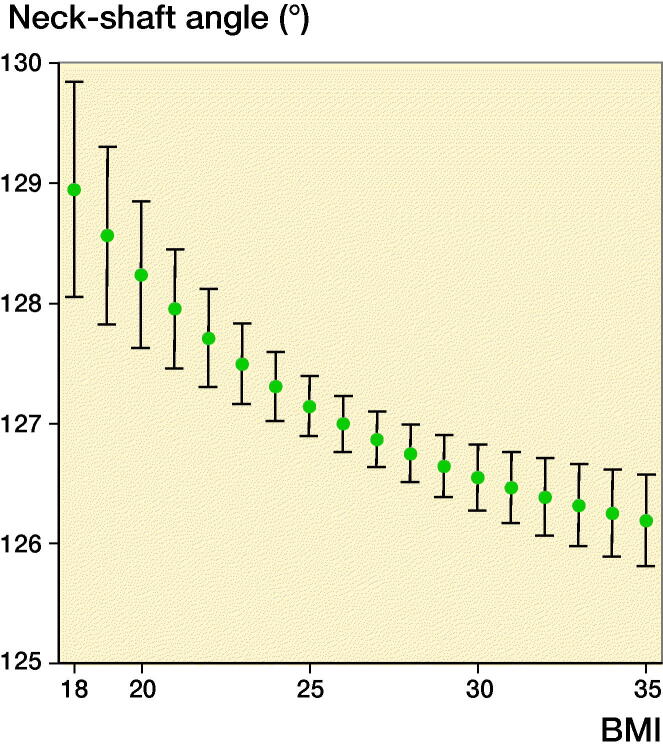
The negative association between neck–shaft angle and body mass index is shown.

**Figure 3.**
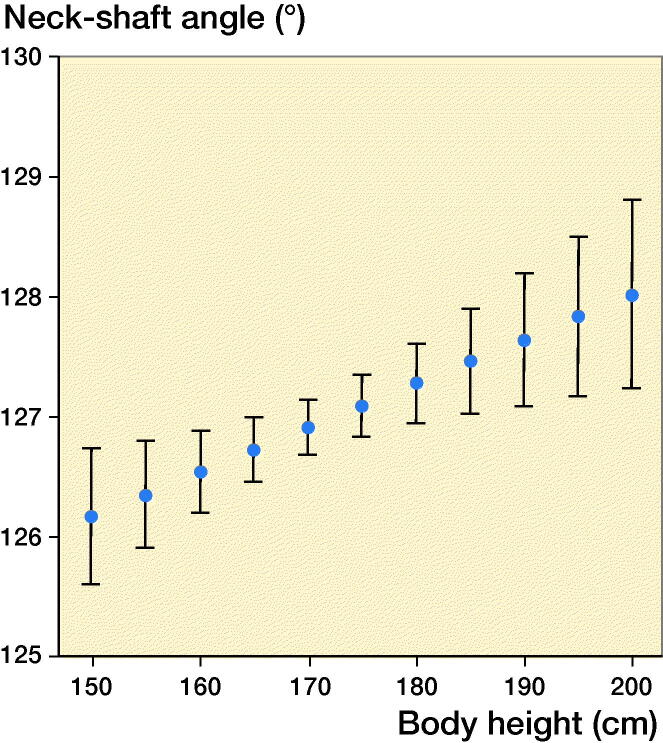
The positive association between neck–shaft angle and body height is shown.

BMI was inversely associated with the NSA in a non-linear fashion (Figure 2, p < 0.001). The decrease in NSA was greater for lower BMI. While body weight was not statistically significant (p = 0.2), body height was (Figure 3, p = 0.004). Per 10 cm of body height, the NSA increased 0.4°. Like BMI, waist circumference was inversely associated with the NSA in a non-linear fashion (p < 0.001). The decrease was more pronounced for smaller than for larger waist circumferences.  

## Discussion

The neck–shaft angle is frequently used for diagnostics, preoperative planning, and therapy (Srisaarn et al. 2019), but no consensus on thresholds or reference ranges exists to date. In addition, various associations with sex and age are described, while other possible associations have rarely been investigated.

Our study may have been limited by the use of non-rotation-corrected coronal MRI images. However, the review by Boese et al. (2016) documented a difference of only 1° between rotation-corrected and non-corrected NSA. Therefore, we suggest that our measurements in coronal MR images are reliable. Nevertheless, due to the 3-D morphology of the femur, it is possible that the NSA has been underestimated. However, previous studies showed similar values for the NSA (Table 3). A second limitation may be the cross-sectional study design, which limits conclusions on cause-and-effect relationships. Thus, further longitudinal studies are necessary. The final limitation may be the possible inclusion of participants with hip pathology. Nonetheless, severe pathology can reasonably be ruled out, because it is mostly treated surgically with metal implants, which should be seen on MRI. Moreover, our sample was a general population, so it could be expected that the majority are healthy. Due to the large sample of the general population, the standardized data acquisition, and the randomized selection process, we assume that reliable population-based data were provided. We found mean NSAs of 126.5° (SD 6.75) for men and 127.4° (SD 6.55) for women. The reference range was 113.9° to 140.0°.

**Table 3.** Neck-shaft angle in other studies

Author, year	Method	Population	Age (range)	n	Mean (SD)
Alonso et al. 2000	DXA	Spain	70	235 M	126.3 (4.4)
			70	310 F	124.6 (4.2)
Doherty et al. 2008	AP radiograph	England	64	1,103	R 128.3 (7.1)
					L 128.3 (7.1)
Kaptoge et al. 2008	DXA	US	74	6,839 F	127.2 (6.0)
Elbuken et al., 2012	DXA	Turkey	(20–108)	18,943	M 129.6
					F 129.1
Bagaria et al. 2012	AP radiograph	India	(20–80)	141 M	127.7 (3.9)
				70 F	126.6 (4.8)
Gilligan et al. 2013	Femora	–	–	8,271	126.4 (5.6)
Mitra et al. 2014	AP radiograph	Iran	35 (24–42)	100 M	R 127.5 (5.3)
					L 127.6 (5.6)
			44 (28–57)	100 F	R 125.4 (6.0)
					L 126.6 (6.3)
Boese et al. 2015	CT, coronal Reconstruction (femoral neck plane)	Germany	53 (18–89)	200 M	129.6 (5.9)
			54 (18–100)	200 F	131.9 (6.8)
					R 130.8 (6.7)
					L 130.8 (6.3)
Dimitriou et al. 2016	3D CT	US	47 (31–58)	61	R 126.7 (4.8)
					L 126.6 (4.5)
Present study	MRI coronal plane	Germany	53 (21–90)	1,587 M	126.5 (6.8)
			52 (21–88)	1,639 F	127.4 (6.6)

DXA = Dual-energy X-ray absorptiometry, M = male, F = female, R = right hip, L = left hip

Various results for the association with age have been described (Nissen et al. 2005, Elbuken et al. 2012). Our study showed a decrease of NSA with age in both sexes, which confirms this association in a general population and supports results of previous studies (Beall et al. 2008, Boese et al. 2015). An explanation for this association might be decreasing bone mineral density and physiological changes. We detected a difference between sides with higher NSA for the right hip. This could be explained by the fact that most people have a dominant right side, but since various results on side differences have been reported (Doherty et al. 2008, Gilligan et al. 2013, Boese et al. 2015, Dimitriou et al. 2016), it could also support the thesis of individual side asymmetry (Anderson and Trinkaus 1998). Regarding the widely discussed differences in sex (Nissen et al. 2005, Elbuken et al. 2012, Gilligan et al. 2013, Mitra et al. 2014), we assessed higher NSA in females confirming previous results (Boissonneault et al. 2014, Boese et al. 2015). Moreover, we found a positive association with body height on the NSA, as did Nissen et al. (2005). Body weight was not associated with NSA, but a negative association with BMI and waist circumference was detected. Therefore, previous results regarding the BMI (Elbuken et al. 2012, Mitra et al. 2014) were confirmed. Interestingly, the decrease in NSA was stronger for lower BMI.

Regarding normal values, Boese et al. (2015) described mean NSA on rotation-corrected CT images of 130.8° (SD 6.49) for the femoral neck plane and 133.6° (SD 6.81) for the anterior pelvic plane. Nevertheless, several studies with large sample sizes report values similar to ours (Alonso et al. 2000, Kaptoge et al. 2008, Bagaria et al. 2012, Boissonneault et al. 2014, Mitra et al. 2014). Furthermore, a review of 21 studies showed a mean NSA of 128.8° (Boese et al. 2016). However, varying reference values between 120° and 140° have been described (Beall et al. 2008, Dolan et al. 2011, Boese et al. 2015). Given that these thresholds were mostly adopted by older studies (Lequesne et al. 1964, Tönnis and Heinecke 1999), and considering changed lifestyles since then, our study provides up-to-date reference values. Our results support the historical upper limit of 140° as the physiological benchmark for general populations. Our lower limit is about 6° lower than the previously used limit of 120°, so the radiological prevalence of hip pathology might have been overestimated. Since associations with sex and age are frequently described, sex- and age-adjusted reference values for the NSA may be more accurate for diagnostics, preoperative planning, and orthopedic surgery.

In conclusion, we support the historical threshold of the NSA regarding the upper limit of 140° but propose a lower limit of 114° based on a general population of adults.

## References

[CIT0001] Alonso C G, Curiel M D, Carranza F H, Cano R P, Perez A D. Femoral bone mineral density, neck–shaft angle and mean femoral neck width as predictors of hip fracture in men and women: Multicenter Project for Research in Osteoporosis. Osteoporos Int 2000; 11(8): 714–20. 11095176

[CIT0002] Anderson J Y, Trinkaus E. Patterns of sexual, bilateral and interpopulational variation in human femoral neck–shaft angles. J Anat 1998; 192(2): 279–85. 9643428 10.1046/j.1469-7580.1998.19220279.x PMC1467761

[CIT0003] Bagaria V, Deshpande S, Kuthe A, Rasalkar D D, Paunipagar B K, Madhugiri T S. Radiographic study of the hip joint to determine anthropometric parameters for Indian population. Eur J Radiol 2012 81(2): 312–16. 21255953 10.1016/j.ejrad.2010.12.037

[CIT0004] Beall D P, Martin H D, Mintz D N, Ly J Q, Costello R F, Braly B A, Yoosefian F. Anatomic and structural evaluation of the hip: a cross-sectional imaging technique combining anatomic and biomechanical evaluations. Clin Imaging 2008; 32(5): 372–81. 18760725 10.1016/j.clinimag.2008.01.026

[CIT0005] Boese C K, Jostmeier J, Oppermann J, Dargel J, Chang D, Eysel P, Lechler P. The neck shaft angle: CT reference values of 800 adult hips. Skeletal Radiol 2015; 45(4): 455–63. 26695396 10.1007/s00256-015-2314-2

[CIT0006] Boese C K, Dargel J, Oppermann J, Eysel P, Scheyerer M J, Bredow J, Lechler P. The femoral neck–shaft angle on plain radiographs: a systematic review. Skeletal Radiol 2016; 45(1): 19–28. 26305058 10.1007/s00256-015-2236-z

[CIT0007] Boissonneault A, Lynch J A, Wise B L, Segal N A, Gross K D, Murray D W, Nevitt M C, Pandit H G. Association of hip and pelvic geometry with tibiofemoral osteoarthritis: multicenter osteoarthritis study (MOST). Osteoarthr Cartilage 2014; 22(8): 1129–35. 10.1016/j.joca.2014.06.010 PMC4195737 24971867

[CIT0008] Coskun Benlidayi I, Guzel R, Basaran S, Aksungur E H, Seydaoglu G. Is coxa valga a predictor for the severity of knee osteoarthritis? A cross-sectional study. Surg Radiol Anat 2015; 37(4): 369–76. 25113012 10.1007/s00276-014-1359-6

[CIT0009] Dimitriou D, Tsai T, Yue B, Rubash H E, Kwon Y, Li G. Side-to-side variation in normal femoral morphology: 3D CT analysis of 122 femurs. Orthop Traumatol Surg Res 2016; 102(1): 91–7. 26867707 10.1016/j.otsr.2015.11.004

[CIT0010] Doherty M, Courtney P, Doherty S, Jenkins W, Maciewicz R A, Muir K, Zhang W. Nonspherical femoral head shape (pistol grip deformity), neck shaft angle, and risk of hip osteoarthritis: a case-control study. Arthritis Rheum 2008; 58(10): 3172–82. 18821698 10.1002/art.23939

[CIT0011] Dolan M M, Heyworth B E, Bedi A, Duke G, Kelly B T. CT reveals a high incidence of osseous abnormalities in hips with labral tears. Clin Orthop Relat Res 2011; 469(3): 831–8. 20886325 10.1007/s11999-010-1539-6 PMC3032877

[CIT0012] Elbuken F, Baykara M, Ozturk C. Standardisation of the neck–shaft angle and measurement of age-, gender- and BMI-related changes in the femoral neck using DXA. Singapore Med J 2012; 53(9): 587–90. 23023899

[CIT0013] Fischer C S, Kuhn J, Ittermann T, Schmidt C, Gumbel D, Kasch R, Frank M, Laqua R, Hinz P, Lange J. What are the reference values and associated factors for center–edge angle and alpha angle? A population-based study. Clin Orthop Relat Res 2018; 476(11): 2249–59. 30024461 10.1097/CORR.0000000000000410 PMC6259987

[CIT0014] Gilligan I, Chandraphak S, Mahakkanukrauh P. Femoral neck–shaft angle in humans: variation relating to climate, clothing, lifestyle, sex, age and side. J Anat 2013; 223(2): 133–51. 23781912 10.1111/joa.12073 PMC3724207

[CIT0015] Kaptoge S, Beck T J, Reeve J, Stone K L, Hillier T A, Cauley J A, Cummings S R. Prediction of incident hip fracture risk by femur geometry variables measured by hip structural analysis in the study of osteoporotic fractures. J Bone Miner Res 2008; 23(12): 1892–1904. 18684092 10.1359/JBMR.080802 PMC2686919

[CIT0016] Lequesne M, Lemoine A, Massare C. Le “complet” radiographique coxofémoral: Dépistage et bilan préopératoire des vices architecturaux de la hanche. J Radiol Electrol Med Nucl 1964; 45:27–44. 14134630

[CIT0017] Mitra A, Khadijeh B, Vida A P, Ali R N, Farzaneh M, Maryam V F, Vahid Y. Sexing based on measurements of the femoral head parameters on pelvic radiographs. J Forensic Leg Med 2014; 23: 70–5. 24661710 10.1016/j.jflm.2014.01.004

[CIT0018] Morvan J, Bouttier R, Mazieres B, Verrouil E, Pouchot J, Rat A, Guellec D, Guillemin F, Coste J, Saraux A. Relationship between hip dysplasia, pain, and osteoarthritis in a cohort of patients with hip symptoms. J Rheumatol 2013; 40(9): 1583–9. 23858046 10.3899/jrheum.121544

[CIT0019] Nissen N, Hauge E M, Abrahamsen B, Jensen, J E B, Mosekilde L, Brixen K. Geometry of the proximal femur in relation to age and sex: a cross-sectional study in healthy adult Danes. Acta Radiol 2005; 46(5): 514–18. 16224928 10.1080/02841850510021562

[CIT0020] Srisaarn T, Salang K, Klawson B, Vipulakorn K, Chalayon O, Eamsobhana P. Surgical correction of coxa vara: evaluation of neck shaft angle, Hilgenreiner-epiphyseal angle for indication of recurrence. J Clin Orthop Trauma 2019; 10(3): 593–8. 31061596 10.1016/j.jcot.2018.06.009 PMC6494758

[CIT0021] Tönnis D, Heinecke A. Acetabular and femoral anteversion: relationship with osteoarthritis of the hip. J Bone Joint Surg Am 1999; 81(12): 1747–70. 10608388 10.2106/00004623-199912000-00014

[CIT0022] Völzke H, Alte D, Schmidt C O, Radke D, Lorbeer R, Friedrich N, Aumann N, Lau K, Piontek M, Born G, Havemann C, Ittermann T, Schipf S, Haring R, Baumeister S E, Wallaschofski H, Nauck M, Frick S, Arnold A, Jünger M, Mayerle J, Kraft M, Lerch M M, Dörr M, Reffelmann T, Empen K, Felix S B, Obst A, Koch B, Gläser S, Ewert R, Fietze I, Penzel T, Dören M, Rathmann W, Haerting J, Hannemann M, Röpcke J, Schminke U, Jürgens C, Tost F, Rettig R, Kors J A, Ungerer S, Hegenscheid K, Kühn J, Kühn J, Hosten N, Puls R, Henke J, Gloger O, Teumer A, Homuth G, Völker U, Schwahn C, Holtfreter B, Polzer I, Kohlmann T, Grabe H J, Rosskopf D, Kroemer H K, Kocher T, Biffar R, John U, Hoffmann W. Cohort profile: the study of health in Pomerania. Int J Epidemiol 2011; 40(2): 294–307. 20167617 10.1093/ije/dyp394

